# Molecular Characterization of a Novel Splicing Mutation Underlying Mucopolysaccharidosis (MPS) Type VI—Indirect Proof of Principle on Its Pathogenicity

**DOI:** 10.3390/diagnostics10020058

**Published:** 2020-01-21

**Authors:** Maria Francisca Coutinho, Marisa Encarnação, Liliana Matos, Lisbeth Silva, Diogo Ribeiro, Juliana Inês Santos, Maria João Prata, Laura Vilarinho, Sandra Alves

**Affiliations:** 1 Research and Development Unit, Department of Human Genetics, INSA, 4000-055 Porto, Portugal; francisca.coutinho@insa.min-saude.pt (M.F.C.); marisa.encarnacao@insa.min-saude.pt (M.E.); liliana.matos@insa.min-saude.pt (L.M.); lisbeth.silva@insa.min-saude.pt (L.S.); diogo.ribeiro@insa.min-saude.pt (D.R.); juliana.santos@insa.min-saude.pt (J.I.S.); laura.vilarinho@insa.min-saude.pt (L.V.); 2 Center for the Study of Animal Science, CECA-ICETA, University of Porto, Praça Gomes Teixeira, 55142, 4051-401 Porto, Portugal; 3 Newborn Screening, Metabolism and Genetics Unit, Department of Human Genetics, INSA, 4000-055 Porto, Portugal; 4 Biology Department, Faculty of Sciences, University of Porto, 4150-179 Porto, Portugal; mprata@ipatimup.pt; 5 i3S—Health Research and Innovation Institute, University of Porto, 4200-135 Porto, Portugal

**Keywords:** mucopolysaccharidosis type VI (MPS VI), lysosomal storage disorders (LSDs), next-generation sequencing (NGS), splicing mutation, functional studies

## Abstract

Here, we present the molecular diagnosis of a patient with a general clinical suspicion of Mucopolysaccharidosis, highlighting the different tools used to perform its molecular characterization. In order to decrease the turnaround time for the final report and contribute to reduce the “diagnostic odyssey”, which frequently afflicts affected families, the proband’s sample was simultaneously screened for mutations in a number of lysosomal function-related genes with targeted next-generation sequencing (NGS) protocol. After variant calling, the most probable cause for disease was a novel *ARSB* intronic variant, c.1213+5G>T [IVS6+5G>T], detected in homozygosity. In general, homozygous or compound heterozygous mutations in the *ARSB* gene, underlie MPS type VI or Maroteaux-Lamy syndrome. Still, even though the novel c.1213+5G>T variant was easy to detect by both NGS and Sanger sequencing, only through indirect studies and functional analyses could we present proof of principle on its pathogenicity. Globally, this case reminds us that whenever a novel variant is detected, its pathogenicity must be carefully assessed before a definitive diagnosis is established, while highlighting alternative approaches that may be used to assess its effect in the absence RNA/cDNA sample(s) from the proband. This is particularly relevant for intronic variants such as the one here reported. Special attention will be given to the use of reporter minigene systems, which may be constructed/designed to dissect the effect of this sort of alterations, providing an insight into their consequences over the normal pre-mRNA splicing process of the affected gene.

## 1. Introduction

Lysosomal Storage Disorders (LSDs) are sometimes challenging to diagnose due to the wide spectrum of phenotypes that the patients may present [1]. Indeed, the recognition of LSDs clinical features requires clinical expertise, as most of them are not specific and can be caused by defects in other metabolic pathways (mitochondrial and peroxisomal) [1,2,3,4]. Therefore, it is common to have a long period between the onset of the first symptoms and the definitive diagnosis. Moreover, even in the presence of typical clinical signs and symptoms, samples and diagnostic tests are different for each group of lysosomal disorders, being often specific of a given disease [1,4]. This is one of the major reasons why definitive diagnosis of LSDs requires a close collaboration between laboratory specialists and clinicians, in order to reach a correct diagnosis in the shortest time. Biochemical genetic testing (BGT) in particular, which includes the determination of the enzymatic activity of lysosomal hydrolases, is feasible for most LSDs and essential for the diagnosis of primary lysosomal enzyme deficiency. In fact, the majority of patients is initially screened by enzyme assays and only after that the molecular studies are performed to determine the disease-causing mutation(s). In families with an already identified causative molecular defect, mutation analysis may be performed directly. Mutation analysis is done through molecular genetic testing (MGT), which can be performed either on DNA or RNA and comprises a range of different approaches for investigating the entire gene-coding regions and exon-intron boundaries, as well as the 5’ and 3´ untranslated regions (UTRs). It is not only useful but frequently necessary to confirm the enzymatic diagnosis of LSDs. For LSDs resulting from non-enzymatic lysosomal proteins, MGT is absolutely essential for a proper diagnosis [2]. Additionally, it is the identification of mutations in the proband and its family that allows for accurate, rapid and reliable MGT for other family members, including prenatal diagnosis. Naturally, the pathological significance of a novel variant has to be investigated before its application to diagnosis and counseling [3].

Whatever the case, MGT has not been used extensively as the primary diagnostic test for LSDs but this paradigm may be about to change thanks to the advent of the so-called next-generation sequencing (NGS), with its unprecedented throughput, scalability and speed [5]. This technology allows the sequencing of either the whole genome/exome or that of selected panels of genes, through a process known as targeted sequencing. Targeted sequencing in particular is now implemented in many labs and this technology is actively speeding up the solution of several diagnostic problems in the LSDs field, as demonstrated by several teams [6,7,8,9,10,11,12,13,14,15,16,17,18]. While being well known that with this unparalleled capacity, NGS is speeding up molecular diagnostics, it is also true that whenever a novel variant is detected, its pathogenicity must be carefully assessed. Furthermore, most specialist laboratories are still working with more traditional methods. Recently published statistics state that currently about 85–90% of LSDs cases are still diagnosed by demonstrating a deficiency of the activity of a lysosomal enzyme [3]. Finally, even for the laboratories where NGS technology is already available, it should always be considered whether a targeted panel will be the most obvious choice, as there are several preliminary screens that can point out, if not the individual disease, at least the subgroup to which it belongs.

For example, for the Mucopolysaccharidoses (MPS) subgroup, the preliminary screen in urine is a routine test that should always be considered. There are 11 different MPSs, resulting from deficiencies in any of the enzymes involved in the stepwise degradation of glycosaminoglycans (GAGs). Deficiencies in each of those enzymes result in different disorders, all sharing a series of clinical features, though in variable degrees: MPS I (OMIMs #607014; # 607015; # 607016); MPS II (#309900); MPS IIIA (#252900), IIIB (#252920), IIIC (#252930) and IIID (#252940); MPS IV A (#253000) and IV B (#253010); MPS VI (#253220); MPS VII (#253200) and MPS IX (#601492). Typical symptoms include organomegally, dysostosys multiplex and coarse facies. Central nervous system (CNS), hearing, vision and cardiovascular function may also be compromised [19].

Traditionally, MPSs are recognized through analysis of urinary GAGs. Over the years, several methods have been devised to allow both qualitative identification and quantitative measurements of the accumulated subtrate(s) [20,21,22,23,24]. Still, initial screenings of urinary GAGs allow discrimination between broad classes of MPSs but cannot distinguish subgroups. In fact, as happens with many other LSDs, complete and definitive diagnosis may only be accessed through a combination of enzymatic assays and molecular analyses. Currently, there are countless specialist laboratories in Europe where those biochemical and genetic tests are carried out. Nevertheless, developing countries often lack the necessary resources/expertise for proper laboratory diagnosis of this type of rare genetic diseases. Being one of the laboratories where MGT for virtually all LSDs (MPSs included) is available for research purposes, we receive several samples from developing countries, whose clinicians and/or centers struggle to get a molecular characterization of affected individuals. Some of our results concerning those studies have already been published [25]. Still, every now and again, a case pops up to highlight how tricky and delicate this process can be, especially in non-optimal circumstances, i.e., without knowing the patient’s clinical picture or assessing their biochemical study and in the absence of proper or sufficient sample(s).

Here, we present our results on the molecular characterization of one of those cases: a patient with a general clinical suspicion of MPS that we have received from Tunisia. Initial studies were performed in gDNA, by NGS with in a custom gene panel for LSDs that includes 86 genes implicated in lysosomal function. After variant calling, a novel homozygous mutation was detected in the *ARSB* gene, encoding arylsulfatase B, the enzyme deficient in MPS VI (Maroteaux-Lamy syndrome): c.1213+5G>T (IVS6+5G>T). Additional analyses included segregation studies through classical sequencing, cDNA sequencing of the *ARSB* gene of the proband’s father, and functional analysis based on reporter minigenes. Altogether, these methods allowed for the full molecular characterization of the novel mutation here described and the indirect assessment of its effect on the normal *ARSB* pre-mRNA splicing.

## 2. Materials and Methods

### 2.1. Samples

Here, we studied a female patient from Tunisia with clinical suspicion of MPS. No further information was provided. Informed consent for genetic research was obtained from the individual guardians. Biological samples from healthy donors were also analyzed. The studies were conducted in agreement with the Declaration of Helsinki and approved by the Ethics Committee of Instituto Nacional de Saúde Dr. Ricardo Jorge (2015DGH1062 and 2016DGH1312), where biological samples were obtained.

### 2.2. Next-Generation Sequence Analysis

Genomic DNA was automated extracted and purified from total blood on a BioRobot EZ1 instrument (QIAGEN, Germantown, MD, USA), using the EZ1 DNA Blood 350 µL Kit (QIAGEN). An NGS-targeted panel covering exonic regions and intronic flanking regions of 86 genes was used. For library preparation, 500 ng of the previously extracted gDNA were used, according to the manufacture protocol (Agilent, Santa Clara, CA, USA). The step-by-step capture protocol is available online (Sureselect QXT Targeted Enrichment for Illumina Multiplexed Sequencing Protocol). The pool was prepared, denaturated, diluted and the sequencing was performed on an Illumina MiSeq platform according to the manufacture protocol for paired-end 150 base pair reads.

Custom-panel design for NGS was prepared with Sure Design software (Agilent Technologies) for the Targeted Enrichment System and Illumina platform based on the last genome build available (*H. sapiens*, hg19, GRCh37, 02.2009) and by selecting for a 150 base-pair read length. The target parameters were the coding exons and the UTRs, including a region extension of 25 bases from the 3′ end and 25 bases from the 5′ end (based on RefSeq database). The stringency parameter was selected to maximize the coverage. The MiSeq Reporter software (Illumina, San Diego, CA, USA) was used for sample demultiplexing and FASTQ file generation. Alignment and variant calling were performed using Surecall (Agilent); variant annotation, on the other hand, was performed using wANNOVAR (http://wannovar.wglab.org/).

### 2.3. Molecular Diagnosis

PCR amplification of *ARSB* exons and adjacent intronic regions, as well as from the promoter region of the gene, was done with specific primers. Each PCR reaction was carried out with specific primers using approximately 40 ng of genomic DNA, 1X the PCR reaction mix ImmoMix™ Red (Bioline, London, UK) and 0.25 µM of each primer (primer sequences and PCR annealing temperatures available on Supplementary Table S1). Thereafter, the samples were heated to 95 °C for 7 min, followed by 35 cycles of denaturation, annealing and extension. The final extension was completed by 5 min at 72 °C.

The amplified fragments were purified with illustra ExoStar™ 1-Step (GE Healthcare Life Sciences, Buckinghamshire, UK) and sequenced using a BigDye Terminator Cycle Sequencing Kit (Applied Biosystems, Foster City, CA, USA) on an ABI PRISM 3130xl Genetic Analyser (Applied Biosystems). Results were analyzed with the sequence analysis software FINCHTV, version 1.3.1. Sequencing profiles were compared with the *ARSB* reference sequence ENST00000264914.8 (https://www.ensembl.org/index.html) using the Clustal Omega multiple sequence alignment bioinformatic tool (https://www.ebi.ac.uk/Tools/msa/clustalo/).

The presence of the novel mutation was confirmed in two independent experimental assays by Sanger sequencing. Additionally, 50 healthy donors were screened for the newly identified *ARSB* mutation trough gDNA sequencing.

### 2.4. RT-PCR Analysis

Total cellular RNA was isolated from blood using the PAXgene Blood RNA System (QIAGEN), and reverse transcribed using the Ready-To-Go You-Prime First-Strand Beads (GE Healthcare Life Sciences). The *ARSB* cDNA was then amplified with specific primers (Supplementary Table S1). Amplification reactions were performed in a total volume of 25 μL containing 12.5 μL of 1X ImmoMix™ Red (Bioline), 2.5 to 5 μL of cDNA and 0.5 μM of each primer. After an initial period of 7 min at 95 °C to activate the Taq polymerase and denaturation of 3 min at 94 °C, the reaction continued for 35 cycles at 94 °C for 30 s, 45 s at annealing temperature, extension at 72 °C for 60 s and a final extension for 10 min at 72 °C.

### 2.5. Bioinformatic Analysis

In order to better understand the potential effect of the novel mutation over to the pre-mRNA splicing, the Human Slicing Finder (HSF)—version 3.1 bioinformatic software (http://www.umd.be/HSF/index.html) was used. The HSF system combines 12 different algorithms to identify and predict mutations’ effect on splicing motifs including the acceptor and donor splice sites, the branch point and auxiliary sequences known to either enhance or repress splicing: Exonic Splicing Enhancers (ESEs) and Exonic Splicing Silencers (ESSs). These algorithms are based on either PWM matrices, Maximum Entropy principle or Motif Comparison method. Overall, this website incorporates data from other well-known bioinformatics tools which may be individually used to evaluate the potential effect of novel mutations such as MaxEntScan [26], ESEfinder [27] and Rescue-ESE [28], while integrating new matrices to identify hnRNP A1, Tra2-β and 9G8 [29].

### 2.6. Minigene Construction

For the splicing analysis of the novel *ARSB* variant [c.1213+5G>T (IVS6+5G>T)], wild-type (WT) and mutant minigene reporter vectors were constructed. Patient and control genomic DNA including 250 bp of the intron 5, the whole exon 6 and 245 bp of intron 6, upstream the variant were amplified. A *XhoI* (Forward primer: 5′ ATA TAT CTC GAG CCA GAA TCA TTT TTT AAA 3′) and *BamHI* (Reverse primer: 5′ ATA TAT GGA TCC GAG AGT TCA ATG GCT TAA 3′) restriction enzyme cleavage site was added to each oligonucleotide. The fresh products were then subcloned into the TOPO vector (Invitrogen, Carlsbad, CA, USA), according to the manufactor’s guidelines. The inserts were restricted with *XhoI* and *BamHI* and after band excision, subsequently purified with Wizard SV Gel and PCR Clean-Up system (Promega, Madison, WI, USA). Then, using the Rapid Ligation Kit (Roche Applied Science, Indianapolis, IN, USA), the inserts were cloned into the pSPL3 vector (Exon Trapping System, Life Technologies, Gibco, NY, USA). All clones were verified by DNA sequencing.

### 2.7. In Vitro Splicing Assays

For the functional splicing assays, Hep3B cells were seeded in 6-well plates (3 × 10^5^) and 24 h later transfected with either WT or mutant minigenes (2 µg) using Lipofectamine 2000 (Invitrogen) according to the manufacturer’s instructions. In this study, 24 h post-transfection cells were collected, total RNA was extracted and cDNA synthesized for subsequent amplification with vector-specific primers (SD6 and SA2—sequences available upon request). The amplified products were separated by agarose gel electrophoresis and purified either after band excision (as described in the previous section) or directly by using illustra ExoStar™ 1-Step (GE Healthcare). Purified amplicons were directly sequenced in an ABI Prism 3130xl genetic analyzer (Applied Biosystems).

### 2.8. Mutation Nomenclature

The Ensembl sequence ENST00000264914.8 of *ARSB* gene was used as reference for residue numbering. Mutation nomenclature followed the guidelines and recommendations of the Human Genome Variation Society (http://varnomen.hgvs.org/) [30].

## 3. Results

### 3.1. Next-Generation Sequencing

The complete list of variants identified in the studied patient using the NGS-targeted panel is presented on Supplementary Table S2. In summary, after variant annotation and filtering steps on the LSDs customized panel, two non-polymorphic variants were detected in MPS-related genes: a known *IDUA* missense mutation in heterozygosity (c.G1225C; p.G409R) [31] and a novel homozygous variant affecting the +5 position of *ARSB* intron 6 (c.1213+5G>T; IVS6+5G>T). Taking into account that both MPS I (caused by mutations in the IDUA gene) and MPS VI (caused by mutations in the *ARSB* gene) are autosomal recessive diseases, and no other pathogenic mutations were detected in heterozygosity in the *IDUA* gene, the most probable cause for disease seemed to be the novel *ARSB* intronic variant. Still, a number of studies had yet to be done in order to assess its pathogenicity.

Concerning the implementation of the NGS technology and the reliability of the obtained sequences, the mean per-base coverage depth for the designed panels was 260-fold, and 97% of the bases were covered by more than 20-fold.

### 3.2. gDNA Screening of the Proband, Segregation Studies and In Silico Analysis of the Newly Identified VUS (c.1213+5G>T [IVS6+5G>T])

**Figure 1:**
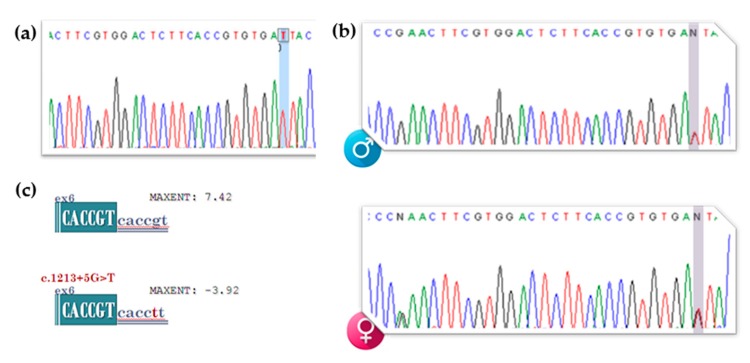
Molecular diagnosis of the patient by classical gDNA screening of the *ARSB* gene: detection of the novel c.1213+5G>T (IVS6+5G>T) mutation. Electropherogram highlighting the affected residue (**a**) in the patient and (**b**) parents. (**c**) *In silico* predictions on the effect of the novel mutation over the splice-site junctions. Score predictions by the MaxEntScan bioinformatic tool.

After classical sequencing of the *ARSB* gene, the presence of the novel c.1213+5G>T (IVS6+5G>T) variant was confirmed in homozygosity (Figure 1a). Apart from this novel variant of uncertain significance (VUS), only non-pathogenic single nucleotide polymorphism (SNPs) were detected.

Targeted segregation studies, which consisted on the amplification of exon 6 and its flanking intronic regions on gDNA samples from the proband’s parents, confirmed the presence of the IVS6(+5) VUS in heterozygosity (Figure 1b). Finally, *in silico* tools also predicted this VUS to be pathogenic, by disrupting the normal 5′ constitutive splice site of exon 6. Particularly, the MaxEntScan splicing score for the 5′ constitutive wt splice site (7.42) was much higher than that predicted when the mutant T was inserted in the +5 position (−3.92; −152.7% variation), clearly pointing to a direct effect over the normal *ARSB* pre-mRNA splicing (Figure 1c). Ideal MaxEntScan splice-site scores are: 11.81 (5′ss) and 13.59 (3′ss). Concerning the predictions on the effect of this VUS over the sequence’s constitutive enhancer motifs, the ESE finder matrices predicted that the mutant T inserted in the +5 position would severely affect a constitutive ESE site for SRSF6 (SRp55), while generating two novel potential ESE sites, for SRSF2 (SC35) and SRSF5 (SRp40).

Finally, the mutation was not found in gnomAD, a genome aggregation database that spans 125,748 exome sequences and 15,708 whole-genome sequences from unrelated individuals sequenced as part of various disease-specific and population genetic studies. The mutation was also not listed in the 1000 genome nor in the 5000 exome.

### 3.3. cDNA Screening and Splicing Pattern Analysis

**Figure 2:**
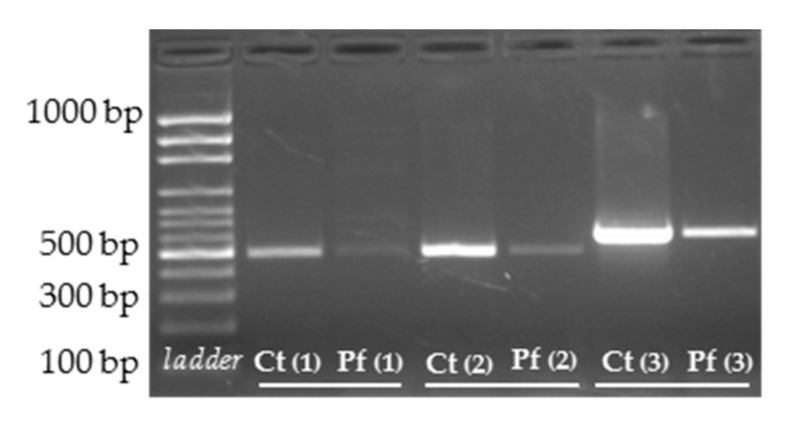
RT-PCR amplification of RNA extracted from the proband’s father (Pf), carrier of the novel c.1213+5G>T [IVS6+5G>T] mutation. Total *ARSB* cDNA was amplified in three different fragments (1–3). The amplification of a control sample (Ct) is also presented.

In order to assess the effect of the VUS under analysis, a sample of the proband was requested for RNA extraction and subsequent reverse transcription into cDNA. It was not possible to establish a skin biopsy for fibroblast culture. Therefore, RNA was extracted from total blood of the proband’s father. The *ARSB* cDNA was then amplified with specific primers and, surprisingly, its transcript pattern was similar to that observed for a control (Figure 2).

### 3.4. Indirect Proof of Principle on c.1213+5G>T [IVS6+5G>T] Pathogenicity

**Figure 3:**
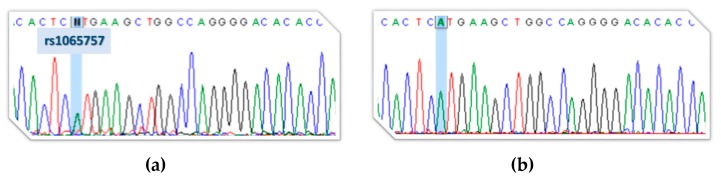
Indirect proof of principle on c.1213+5G>T [IVS6+5G>T] pathogenicity. Electropherograms highlighting the rs1065757 polymorphism (G>A) in the proband’s father (Pf) gDNA (heterozygous; (**a**)) and cDNA (homozygous A; (**b**)). The discrepant pattern observed for the exonic polymorphism (rs1065757, c.1072G>A) provides indirect proof of an active nonsense-mediated mRNA decay (NMD) mechanism causing the degradation of the allele harboring the splicing mutation.

After conducting a classical sequencing approach of the *ARSB* gDNA and cDNA on the proband’s father, a discrepant pattern was observed for the exonic rs1065757 polymorphism (c.1072G>A). Even though the individual was shown to be heterozygous for that SNP at gDNA level, when his cDNA was sequenced after RT-PCR, he seemed to be homozygous for the less common A allele rs1065757 (Figure 3). This suggests that one allele was most likely degraded, corroborating the *in silico* predictions.

### 3.5. Functional Analyses

**Figure 4:**
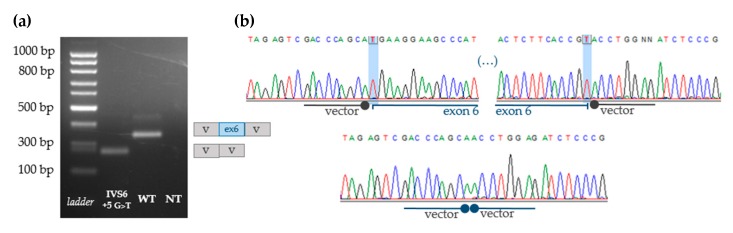
RT-PCR amplification and Sanger sequencing of WT and mutant splicing reporter minigenes using vector-specific primers. The results of RT-PCR analysis using vector specific primers are shown along with a diagram of the transcripts obtained in Hep3B cells after 24 h of incubation (**a**) and their sequencing results depicted in electropherograms (**b**). c.1213+5G>T [IVS6+5G>T]: mutant minigene; WT: wild-type; NT: non-transfected.

To further study the effect of the novel *ARSB* variant on splicing pattern, minigene assays were performed using constructs generated with the WT or mutant sequences (exon 6 plus its respective intronic flanking regions), in the pSPL3 vector. The results of RT-PCR analysis using vector-specific primers are depicted in Figure 4 and clearly show an effect of the +5 nucleotide substitution under analysis over the correct splicing of exon 6. In fact, while the WT amplicon spans approximately 350 bp, the one referring to the mutated minigene (harboring the c.1213+5G>T [IVS6+5G>T] nucleotide change), has a considerably lower molecular weight with less than 300 bp (Figure 4a). These results were further confirmed by Sanger sequencing. As expected, the WT amplicon revealed a transcript with the inclusion of exon 6 sequence (71 nucleotides). For the c.1213+5G>T [IVS6+5G>T] mutant, however, *ARSB* exon 6 got skipped. Therefore, the sequence resulting from RT-PCR of the mutated minigene, corresponds only to pSPL3 vector (Figure 4b).

## 4. Discussion

After targeted NGS sequencing and variant calling analyses, the novel homozygous variant affecting the +5 position of *ARSB* intron 6 (c.1213+5G>T; IVS6+5G>T) as poped up as the most probable cause for disease. In fact, both bioinformatic predictors and literature support its pathogenecity. On one side the HSF bioinformatic tool predicted the disruption of the WT donor site most probably affecting splicing and, on the other, a bibliographic search of *ARSB* gene variants unveiled the existence of a MPS VI-causing mutation affecting the same residue and altering the normal splicing pattern (c.1213+5G>A [IVS6+5G>A]) [32], clearly pointed to a pathogenic effect over splicing. Actually, the guanine residue at the fifth position of the donor splice site consensus sequence is 75% conserved in humans [33] and any alteration over that residue may hold the potential to affect the normal splicing process. Moreover, segregation studies confirmed its presence in heretozygozity in both parents (Figure 1b). Thus, it seemed quite tempting to assume that this would be the variant underlying the patient’s phenotype by affecting the normal *ARSB* splicing. Still, only after a proper cDNA analysis could we confirm its effect in splicing and, subsequently, its pathogenicity. Unfortunately, we only had access to an extremely degraded cDNA sample obtained from blood of one of her parents. Interestingly, the splicing pattern observed after cDNA amplification of that sample was absolutely normal (Figure 2). Still, taking into account that the original gDNA sample had been screened for mutations in 86 different genes related to lysosomal function and there were no more variants that could justify his phenotype (Supplementary Table S2), there was only one hypothesis that could justify the normal splicing pattern observed in the proband’s father: an active nonsense-mediated mRNA decay (NMD) mechanism causing the degradation of the allele harboring the splicing mutation. However, its pathogenicity was yet to be proven.

Therefore, we conducted a classical sequencing approach of the *ARSB* gDNA and cDNA on the proband’s father and ended up demonstrating that, while being heterozygous for an exonic SNP (rs1065757, c.1072G>A; Figure 3a) in the surroundings of the mutation, the same individual seemed to be homozygous for those exact same SNP (A) at cDNA level (Figure 3b). In accordance with these observations, when the proband’s gDNA sequence was double-checked for the presence of the SNP, it was shown to be wild-type homozygous (G). This observation provided indirect proof of the mutation’s effect on splicing, further suggesting that the mutant transcript(s) is degraded by NMD.

Still, much had yet to be proven concerning the effect of this novel mutation over the normal splicing of *ARSB*. It was, therefore, mandatory to further explore this novel mutation in order to fully characterize its effect at cDNA level. Having no further access to extra samples from either the proband herself or her mother, we designed a strategy to artificially evaluate the impact of this mutation at cDNA level by constructing WT and mutant minigenes to functionally investigate the splicing defects caused by the novel c.1213+5G>T *ARSB* mutation. Briefly, the relevant genomic segment (including exon 6 and its intronic boundaries) was cloned within a plasmid (pSPL3) specifically designed for splicing analysis, since its multiple cloning site is flanked by functional strong splice donor and acceptor sites.

After RT-PCR, it was possible to observe a clear effect of the novel c.1213+5G>T (IVS6+5G>T) variant over exon 6 constitutive splicing. In fact, the RT-PCR amplification product of the mutant minigene was significantly smaller than that seen for the WT minigene (Figure 4a). Direct sequencing of the amplicon further demonstrated the skipping of the full-length exon 6 sequence (71 nucleotides) in the mutant minigene, while those obtained for the WT minigene comprehended 71 nucleotides corresponding to the exon 6 sequence (Figure 4b). Exon 6 skipping results in an alteration of the *ARSB* mRNA open reading frame (ORF) with the introduction of 11 amino acids, which differ from the WT protein, ultimately resulting in the appearance of a premature stop codon (PTC). *In silico* translation of the mutant *ARSB* cDNA sequence results in the generation of a truncated protein with 141 amino acids less than the WT one. If generated, one such protein is not expected to be functional and, therefore, its underlying mutation is clearly pathogenic.

In general, these functional studies allowed us to establish the pathogenic potential of the novel c.1213+5G>T (IVS6+5G>T) mutation. Still, a number of questions may still be raised. For example, it remains unclear whether this particular +5 change promotes the skipping of exon 6 alone or if it affects other exons as well. In fact, bioinformatic tools are clearly not sufficient when it comes to predict the exact effect of a particular change, even though they are usually quite reliable on evaluating whether it does affect splicing or not. For example, if we look at bioinformatic predictions concerning the other pathogenic mutation known to affect the +5 region of exon 6, the intronic mutation c.1213+5G>A [IVS6+5G>A] [32], that alteration would theoretically lead to incorrect splicing of exon 6. However, when the authors analyzed by RT-PCR the cDNA from the patient harboring that mutation, it was shown to produce two different aberrant transcripts: one with a deletion of exon 6 and one with a deletion of exons 5 and 6 [32]. One possible approach to understand whether the same happens with the novel c.1213+5G>T (IVS6+5G>T) mutation would be to design a larger minigene, including exon 5 and/or exon 7, to check whether additional alternative transcripts with further exons skipped would also be observed.

Ultimately, only a direct analysis of the proband’s *ARSB* cDNA and/or protein product could provide complete evidence on the effect of this novel mutation over splicing. Nevertheless, the construction and transient expression of minigenes harboring the mutation under study allowed confirmation of its effect over the splicing process. Furthermore, the results obtained with reporter minigenes add up to those from the *ARSB* transcript analysis of the proband’s father sample. Altogether, the two methods point out to the same general conclusion and provide solid indirect proof on the novel mutation’s pathogenicity, even in the absence of an RNA/cDNA sample from the proband.

## 5. Conclusions

Here, we present the case of a molecular diagnosis of a patient with a general clinical suspicion of MPS.

In summary, the case report here presented is a perfect example on how complex and delicate the whole MGT process can be, even now, in the ‘NGS era’. In fact, even though NGS holds a huge potential as an MGT tool, it is also responsible for the identification of a growing number of VUS in the same sample. Thus, functional studies are growing more and more necessary for proper molecular diagnosis. Minigenes, in particular, may be an interesting tool whenever an effect on splicing is predicted but no RNA/cDNA samples are available.

## Supplementary Materials

The following are available online at https://www.mdpi.com/2075-4418/10/2/58/s1, Table S1: Primer sequences and PCR annealing temperatures for *ARSB* amplification; Table S2: List of variants identified in the studied patient using the NGS-targeted panel.
