# Supplementary material for: Molecular Characterization of a Novel Splicing Mutation Underlying Mucopolysaccharidosis (MPS) Type VI—Indirect Proof of Principle on Its Pathogenicity
Source: Diagnostics (Basel). 2020 Jan 21;10(2):58. doi: 10.3390/diagnostics10020058 (PMC7168280; doi:10.3390/diagnostics10020058)
Supplement: Supplementary file 1 [file diagnostics-10-00058-s001.pdf]

diagnostics

Table S1. Primer sequences and PCR annealing temperatures for *ARSB* amplification.

Primer	Sequence (5′→3′)	Fragment Size (bp)	Annealing Temperature (°C)
**ARSB *gDNA amplification***			
gARSB_prF	CTGTTTGCTAGTGGGGAGGA	595	60
gARSB_prR	CCCCTTGTACCGCTGATAGA		
gARSB_1F	GTTCGTCTCTGGCTCCTCCT	686	58[#]
gARSB_1R	GCCTGGAAGAGCGAGGTT		
gARSB_2F	GAAGGCCATTTTATCTGCTTG	399	60
gARSB_2R	AAAGCAGCCCCATTACAGTG		
gARSB_3F	TAGCCTCGTCACGGGTAATC	382	60
gARSB_3R	CAACAATGGCCTTTTCCTACA		
gARSB_4F	TGCATTCTGTAGGTTGTCTTGA	471	60
gARSB_4R	TCCACAATTACCATGTCTCCA		
gARSB_5F	GGGAAAAGGCAAGGAATTTT	493	60
gARSB_5R	TCATGTATTTGTAAGCTGAACTATCA		
gARSB_6F	TTCAAAGGGTCCCAGAATCA	496	60
gARSB_6R	AGCACACTGCCCTCTGAGAT		
gARSB_7F	TTGCGGTGGTTTATGACTGA	487	60
gARSB_7R	GGTGGGAAACGGTTAGAACA		
gARSB_8F	CCACACCCACAACCCAGT	500	60
gARSB_8R	CCTCGGTGTGGTTTAAGAGC		
**ARSB *cDNA amplification***			
**Primer**	**Sequence** (5′→3′)	**Fragment Size** (bp)	**Annealing Temperature** (°C)
cARSB_1F	GCAGCCCAGTTCCTCATTCT	466	56
cARSB_1R	GGCAGGAGTTTTTCATCCAG		
cARSB_2F	CTGCTCACTGGCCGCTA	587	60
cARSB_2R	GTGTTGTTCCAGAGCCCACT		
cARSB_3F	TCTCCAGTCTGTGCATGAGC	650	60
cARSB_3R	GTGGAGGGAACCAGTAACCA		
cARSB_4F	GCTCCAGCAAAGGATGACTC	395	60
cARSB_4R	GGTTTTCTAGCCTCCCTGAAA		

[#]For some samples, the fragment amplified with this pair of primers may display considerable amounts of unspecific products. Therefore, 5% of betaine and/or 5%DMSO may be added to the reaction mix, whenever necessary.

Table S2. List of variants identified in the studied patient using the NGS-targeted panel.

Gene Name	Variant (RefSeq)	State[1]	Variant Characterization
Exonic Mutations			
ATP13A2	NM_022089:exon28:c.C3335G:p.T1112S	Het	Not described
MTX1	NM_002455:exon7:c.T1051C:p.F351L	Het	Not described as associated to disease
CHIT1	NM_003465:exon5:c.C447T:p.A149A	Het	Not described as associated to disease
IDUA	NM_000203:exon9:c.G1225C:p.G409R	Het	Described as pathogenic

GUSB	NM_000181:exon1:c.C162T:p.N54N	Het	Described as non-pathogenic
PEX1	NM_000466:exon8:c.A1579G:p.T527A	Het	Uncertain significance
HGSNAT	NM_152419:exon2:c.C204T:p.T68T	Hom	Not described as associated to disease
ABCA1	NM_005502:exon46:c.C6083T:p.A2028V		Not described as associated to disease
ABCA1	NM_005502:exon39:c.T5301C:p.Y1767Y	Het	Described as non-pathogenic
MYO7A	NM_000260:exon39:c.T5356A:p.S1786T	Het	Described as non-pathogenic
MYO7A	NM_000260:exon39:c.A5381C:p.E1794A	Het	Not described
CLN5	NM_006493:exon1:c.C234G:p.A78A	Het	Described as non-pathogenic
GALC	NM_000153:exon13:c.C1403G:p.T468S	Hom	Not described as associated to disease
DYM	NM_017653:exon7:c.A526G:p.I176V	Het	Described as non-pathogenic
IDS	NM_000202:exon6:c.C792T:p.Y264Y	Het	Not described

Intronic Mutations

Gene Name	Transcript ID and Variant	State[1]	Variant Characterization
ATP13A2	ENST00000326735.13: c.1306+42_1306+43insC	Hom	Not described as associated to disease
SORT1	ENST00000256637.8: c.2141+53G>A	Het	Not described as associated to disease
SFTPD	ENST00000372292.8: c.550+26G>T	Het	Not described as associated to disease
ACP2	ENST00000672073.1: c.962+40G>A	Het	Not described as associated to disease
ACP2	ENST00000672073.1: c.962+33G>A	Het	Not described as associated to disease
NPC1	ENST00000269228.10: c.3246-20G>A	Het	Described as non-pathogenic
NPC1	ENST00000269228.10: c.220+52G>C	Het	Described as non-pathogenic

UTR Region

Gene	Variant (RefGene)	State[1]	Variant Characterization
SUMF1	NM_182760:c.*4C>T	Het	Not described as associated to disease
PSAP	NM_002778:c.-29C>T	Het	Described as non-pathogenic

[1] Homozgous (Hom) or Heterozygous (Het).

© 2020 by the authors. Submitted for possible open access publication under the terms and conditions of the Creative Commons Attribution (CC BY) license (http://creativecommons.org/licenses/by/4.0/).
